# An evaluation of podiatry service use for people with inflammatory rheumatic diseases: a review of a rheumatology podiatry clinic in Aotearoa New Zealand

**DOI:** 10.1186/s13047-022-00542-7

**Published:** 2022-05-16

**Authors:** Vy Nguyen, Angela Brenton-Rule, Nicola Dalbeth, Keith Rome, Sarah Stewart

**Affiliations:** 1 School of Clinical Sciences, Faculty of Health and Environmental Sciences, Auckland University of Technology, 90 Akoranga Drive, Northcote, Auckland, New Zealand; 2 Active Living and Rehabilitation: Aotearoa New Zealand, Health and Rehabilitation Research Institute, School of Clinical Sciences, Auckland University of Technology, Private Bag 92 006, Auckland, 1142 New Zealand; 3 Department of Medicine, Faculty of Medical and Health Sciences, University of Auckland, 85 Park Road, Grafton, Auckland, 1023 New Zealand

**Keywords:** Podiatry service, Foot problems, Rheumatology

## Abstract

**Background:**

Foot problems, including foot pain, structural deformities, skin and nail lesions, and footwear difficulties, are common in people with inflammatory rheumatic diseases. However, dedicated podiatry services are limited, including in Aotearoa New Zealand. This study aimed to evaluate the podiatry service use for people with inflammatory rheumatic diseases who attended a specialist podiatric rheumatology clinic in Aotearoa New Zealand.

**Methods:**

This retrospective review included people with an inflammatory rheumatic disease who attended the Auckland University of Technology Podiatric Rheumatology Clinic between 2010 and 2021. Data were extracted manually from patients’ clinical records, including variables relating to patient characteristics, appointment details, presenting complaint, assessments performed, and treatments provided.

**Results:**

From 2010 to 2021, 157 people with inflammatory rheumatic diseases attended 1570 appointments. The most common presenting concern was foot pain (reported by *n* = 121, 77.1% patients during at least one appointment), followed by skin/nail lesions (*n* = 98, 62.4%) and footwear/orthotic needs (*n* = 90, 57.3%). A range of podiatric interventions were provided to address foot-care needs, in which education (*n* = 151, 96.2%) and general skin/nail care (*n* = 107, 68.2%) were the most common treatments provided. The majority of patients also received footwear interventions at some point during their period of service provision (*n* = 96, 61.1%), followed by orthoses, other padding/offloading devices, wound care, exercise prescription and referrals to other health professionals.

**Conclusions:**

This is the first study to review podiatric service provision for people with inflammatory rheumatic diseases attending a specialist podiatric rheumatology clinic in Aotearoa New Zealand. The results of this study have shown that a podiatry clinic dedicated to people with inflammatory rheumatic diseases addresses the wide range of foot problems through an extensive provision of treatment services.

**Supplementary Information:**

The online version contains supplementary material available at 10.1186/s13047-022-00542-7.

## Background

Inflammatory rheumatic diseases, including gout, psoriatic arthritis, rheumatoid arthritis, and systemic lupus erythematosus, are characterised by inflammation of joints and surrounding soft tissue, as well as extra-articular and systemic symptoms [1]. Foot and lower limb problems are significantly more prevalent in this population compared to people without inflammatory rheumatic diseases, with foot pain being the most common symptom reported [2] and a major determinant of lower limb disability [3, 4]. Compared to healthy individuals, people with inflammatory rheumatic diseases also present with reduced foot and ankle motion, muscle weakness, and a high prevalence of structural foot deformities [4–6].

The needs of people with inflammatory rheumatic diseases are often complex and foot problems are frequently overlooked during routine assessments in general practice and rheumatology [7, 8]. A range of studies and international guidelines recognise the importance of podiatrists in the interdisciplinary management of people with inflammatory rheumatic diseases in order to relieve pain, maintain function and improve quality of life [9–11]. These guidelines recommend the provision of podiatry services for assessment and periodic review of foot health needs, including appropriate footwear and orthoses for comfort, mobility and stability [12–14]. Unfortunately, dedicated podiatry services directed towards people with inflammatory rheumatic diseases are scarce [15–17], including in Aotearoa New Zealand [18], where 5.8% of older adults aged ≥ 65 years are living with rheumatoid arthritis, and an even higher 11.9% with gout (with Māori and Pacific peoples disproportionately affected by gout) [19]. A 2009 audit in Aotearoa New Zealand revealed that three quarters of people with rheumatoid arthritis who reported disabling foot pain, had not received a foot assessment, nor seen a podiatrist [18].

In support of the need for improved access to podiatry services for people with inflammatory rheumatic diseases, the podiatry school at Auckland University of Technology (AUT) established a Podiatric Rheumatology Clinic in 2010, which remains one of only two podiatry clinics in Aotearoa New Zealand providing dedicated services to people with inflammatory rheumatic diseases. The AUT Podiatric Rheumatology Clinic welcomes both adult and paediatric patients, including those with juvenile idiopathic arthritis. As a clinical teaching environment, the clinic offers a standard approach to service provision consistent with usual practice, where patients are assessed and treated by podiatry students under the supervision of highly experienced podiatric clinicians.

An important step in tailoring specific foot-care assessment and management approaches to people with inflammatory rheumatic diseases, is to improve understanding of the foot-care needs and current services provided to this population. This study aimed to evaluate podiatry service use for people with inflammatory rheumatic diseases who attended the AUT Podiatric Rheumatology Clinic between 2010 and 2021.

## Methods

A total population sampling approach was used to identify all people with an inflammatory rheumatic disease who were coded as a “rheumatology patient” within the AUT Podiatry Clinic business practice software package (Gensolve Practice Manager™) from March 2010 through to the end of December 2021. The AUT Podiatric Rheumatology Clinic is part of a larger inter-disciplinary healthcare centre offering other podiatry services, as well as physiotherapy, occupational therapy, oral health, counselling, and psychotherapy. Referrals to the Podiatric Rheumatology Clinic are primarily from local rheumatologists and general practitioners, however, the Clinic also welcomes within-centre referrals and self-referrals. Depending on their foot-care needs, patients may attend a single appointment at the Podiatric Rheumatology Clinic, or attend regular ongoing appointments. Patients were included if they had a physician-confirmed diagnosis or self-reported physician diagnosis of at least one type of inflammatory rheumatic disease and attended one or more podiatry appointments. Patients were not included in the review if they had a diagnosis of osteoarthritis in the absence of another inflammatory rheumatic disease, or who were referred but did not attend an appointment at the clinic, or had an unknown or unconfirmed diagnosis.

Ethical approval was obtained from the Auckland University of Technology Ethics Committee (21/405). The need to obtain consent from patients for the use of their information, which was collected as part of usual clinical care, was waived by the Ethics Committee.

### Data extraction

Data from clinical records of each appointment attended by all included patients were extracted from the clinic’s business practice software package into a standardised Microsoft Excel form. Extracted data included patient demographic and medical characteristics, appointment details, presenting complaint, assessments performed, and treatments provided. Patient characteristics included age group, gender, ethnicity, rheumatic disorder(s), and disease duration(s). The details of each appointment included the referrer, wait time from referral to podiatry appointment, total podiatry appointments attended, total duration of podiatry service provision, and the average time between appointments (if applicable).

Data related to the patients presenting complaint(s) and the foot problem(s) identified from the podiatric assessments which were undertaken for each patient according to clinical need were extracted. Presenting complaint(s) were categorised as general skin/nail problems, foot pain, footwear/orthotic concerns, back and/or lower limb pain, foot deformity, neurological symptoms (tingling, numbness), wounds/ulcers, falls/balance issues, and ‘other’. Foot problem(s) identified were categorised as skin/nail, vascular, neurological, structural, biomechanical (static and dynamic), footwear/orthotic, falls risk, and ‘other’. ‘Vascular’ problems were considered present if evidence of arterial disease (i.e., abnormal pulses) or features of venous disease (i.e., telangiectasia, varicose veins) were present. Absent pedal hair was considered a part of vascular assessments and was not a vascular problem on its own.

Data related to service provision were also extracted based on the treatment(s) provided. Treatment(s) provided were categorised as education, skin/nail care, orthoses, footwear, wound care, padding/offloading, referral, exercise prescription, and ‘other’. Footwear education in the form of a verbal recommendation or formal footwear prescription was coded as a ‘footwear intervention’ rather than ‘education’. ‘Education’ included advice on moisturising skin, removing dressings, and the importance of ongoing skin/nail care. Otoform™ props, felt pads, toe props, and similar offloading devices were coded under ‘padding/offloading’ rather than ‘orthoses’. Orthotic devices were generally prefabricated, consistent with the recently reported trend among Aotearoa/New Zealand podiatrists [20]. ‘Wound care’ included wound dressings and wound debridement. ‘Referrals’ required evidence of a referral letter uploaded into the clinic’s business practice software package. Verbal advice provided to patients to talk to their general practitioner or other practitioner was not included under ‘referrals’.

All service provision data were coded as ‘present or absent. All biomechanical assessments performed (or foot problems identified during these assessments) with the patient seated or standing still were categorised under ‘biomechanical (static)’. ‘Biomechanical (dynamic)’ referred to any assessments performed (or foot problems identified during these assessments) when the patient was moving (i.e., gait analyses and plantar pressure analyses).

Prior to extraction, a reliability test was conducted in which two authors (VN, SS) extracted data independently from 15 appointments of a randomly selected patient. The percentage agreement between authors across all extracted data was 93.3% (418/448 extracted data points). The authors discussed all disagreements and developed some coding rules to ensure consistency before a single author (VN) completed the remaining data extraction.

### Data analysis

Microsoft Excel was used to analyse the extracted data and calculate descriptive statistics. All categorical data were described as frequencies and percentages, and continuous data were described as mean (SD). Service provision data were described at patient-level, in which the number of patients with the variable present during at least one appointment was used as the denominator. As an additional analysis, appointment-level data were also analysed, in which the total number of appointments across all patients was used as the denominator. The proportion of foot problems identified from the assessments performed was also calculated using both patient and appointment level data. To examine the differences in patient demographic and medical characteristics between patients who attended the AUT Podiatric Rheumatology Clinic and referred patients who did not attend a clinic appointment, Pearson’s Chi-squared and independent t-tests were used for categorical and continuous data, respectively. *P*-values less than 0.05 were considered statistically significant. All inferential analyses were conducted in IBM SPSS Statistics v.27.

## Results

### Patients

**Fig. 1:**
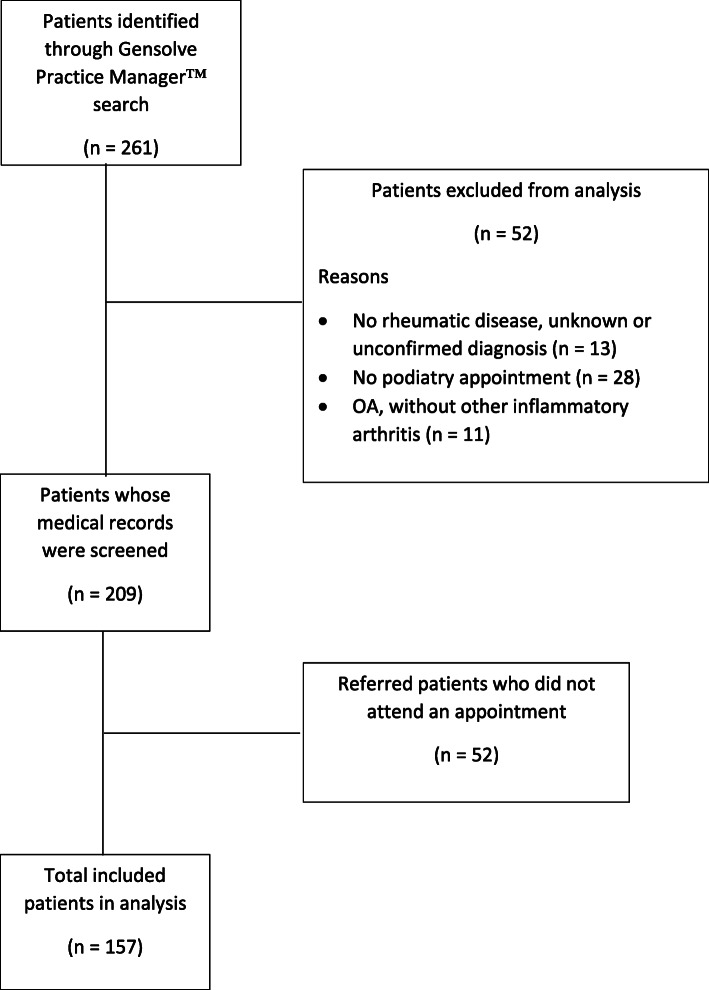
Patient flow chart

There were 261 patients identified as having an inflammatory rheumatic disease under the AUT podiatry clinical records software between 2010 and 2021. Of those, 157 patients met the inclusion criteria and attended at least one appointment (Fig. 1). The baseline patient characteristics for the 157 included patients are summarised in Table 1. The included patients were predominantly female (*n* = 121, 77.1%), New Zealand European ethnicity (*n* = 116, 73.9%), and between the age of 51 and 70 years (*n* = 69, 43.9%). The proportion of Māori (*n* = 5, 3.2%) and Pacific peoples (*n* = 4, 2.6%) was low. Patients had a mean (SD) disease duration of 19.6 (14.6) years, with rheumatoid arthritis being the most common inflammatory rheumatic disease (*n* = 123, 78.3%). There were very few paediatric patients and only 4 (2.6%) with juvenile idiopathic arthritis. Eighty-nine (56.7%) patients were referred by a rheumatologist, followed by self-referrals (*n* = 30, 19.1%). The median wait time from referral to the first podiatry appointment was less than two months (57 days). The mean (SD) total podiatry appointments per patient was 10 (15.2) and total duration of podiatry service provision per patient was 731.3 (1109.8) days. The mean (SD) average time between appointments per patient was 122.9 (188.4) days.

**Table 1:** Baseline patient characteristics for patients who attended at least one appointment at the Podiatric Rheumatology Clinic (*n* = 157 patients)

Age group, years, n (%)	< 10	0 (0.0%)
	11–20	1 (0.6%)
	21–30	5 (3.2%)
	31–40	12 (7.6%)
	41–50	17 (10.8%)
	51–60	29 (18.5%)
	61–70	40 (25.5%)
	71–80	42 (26.8%)
	81–90	11 (7.0%)
	90+	0 (0.0%)
Gender, n (%)	Male	36 (22.9%)
	Female	121 (77.1%)
Ethnicity, n (%)	New Zealand Māori	5 (3.2%)
	Pacific peoples	4 (2.5%)
	New Zealand European	116 (73.9%)
	Asian	22 (14.0%)
	Middle Eastern/Latin American/African	2 (1.3%)
	Not reported	8 (5.1%)
Rheumatic disease^a^, n (%)	Gout	8 (5.1%)
	Juvenile idiopathic arthritis	4 (2.6%)
	Other spondylarthritis	8 (5.1%)
	Psoriatic arthritis	14 (8.9%)
	Rheumatoid arthritis	123 (78.3%)
	Scleroderma	2 (1.3%)
	Systemic lupus erythematosus	7 (4.5%)
	Other	6 (3.8%)
Disease duration, years, mean (SD), range		19.6 (14.6), 0.5–79.0
Referrer, n (%)	Rheumatologist	89 (56.7%)
	Self-referred	30 (19.1%)
	General practitioner	9 (5.7%)
	Other (Physiotherapist, general podiatry clinic)	9 (5.7%)
	Unknown	20 (12.7%)
Wait time (days) from referral to podiatry appointment^b^, median (range)		57.0 (1–2134)
Total podiatry appointments per patient, mean (SD), range		10 (15.2), 1–71
Total duration of podiatry service provision per patient, days, mean (SD), range		731.3 (1109.8), 1–4319
Average time between appointments per patient^c^, days, mean (SD), range		122.9 (188.4), 9–1437

^a^15 patients had 2 rheumatic diseases, and 1 patient had 4 rheumatic diseases

^b^Data available from *n* = 87 patients; ^c^Based on 110 patients with more than one appointment

The review also identified 52 patients who were referred from rheumatologists but did not attend an appointment at the AUT Podiatric Rheumatology Clinic. The characteristics of these 52 patients and the reasons for referral are presented in Additional Table 1. Compared to included patients who attended appointments, there were no differences in gender, ethnicity or age group (all *P* > 0.05). However, patients who did not attend included a lower proportion with rheumatoid arthritis and a higher proportion with other inflammatory rheumatic diseases (*P* < 0.05). Foot pain (*n* = 23, 44.2%), footwear/orthotics (*n* = 22, 42.3%), and foot deformity (*n* = 21, 40.4%) were the main reasons for referral among this group of patients.

### Presenting complaint

Foot pain (*n* = 121, 77.1%) was reported by patients as the most common complaint during at least one appointment, followed by general skin/nail problems (*n* = 98, 62.4%), and footwear/orthotic concerns (*n* = 90, 57.3%) (Table 2).

**Table 2:** Presenting complaints and foot problems identified from podiatry assessments for patients who attended the Podiatric Rheumatology Clinic (*n* = 157 patients)

Presenting complaint^a^, n (%)	Skin/nail problems	98 (62.4%)
	Foot pain	121 (77.1%)
	Footwear/orthotics	90 (57.3%)
	Back and/or lower limb pain	18 (11.5)
	Foot deformity	10 (6.4%)
	Neurological symptoms	11 (7.0%)
	Wounds/ulcers	7 (4.5%)
	Falls/balance issue	23 (14.6%)
	Other	25 (15.9%)
Foot problems identified^b^, n (%)	Skin/nail	126 (80.3%)
	Vascular	68 (43.3%)
	Neurological	66 (42.0%)
	Structural	106 (67.5%)
	Biomechanical (static)	131 (83.4%)
	Biomechanical (dynamic)	101 (64.3%)
	Footwear/orthotic	116 (73.9%)
	Falls risk	3 (1.9%)
	Other	2 (1.3%)

^a^Reported by the patient during at least one appointment

^b^Identified during at least one appointment

Based on appointment-level data, general skin/nail problems were the most common presenting complaints (*n* = 1273, 81.1% appointments) (Additional Table 2).

### Foot problems identified from assessments performed

Based on podiatric clinical assessments, an even larger proportion of patients (*n* = 116, 73.9%) had inappropriate footwear/orthotic devices (Table 2). Skin/nail problems were also commonly identified during clinical examination (*n* = 126, 80.3% patients), as well as musculoskeletal foot problems, relating to structure (*n* = 106, 67.5%), and static and dynamic function (*n* = 131, 83.4%, and *n* = 101, 64.3%, respectively). The proportion of foot problems identified from the performed assessments is presented in Table 3. Biomechanical (static) foot problems were the most common problem identified from the assessments performed (131/138, 94.9% patients), followed by skin/nail problems (126/128, 98.4%). Vascular assessments were performed for 121 (77.1%) patients and 68 (43.3%) patients were identified as having circulation issues (68/121, 56.2%). Although only 11 (7.0%) patients reported neurological symptoms (tingling, numbness), regular neurological assessments were performed for 114 (72.6%) patients with 66 (42.0%) demonstrating neurological impairments (66/114, 57.9%).

**Table 3:** Proportion of foot problems identified from assessments performed (*n* = 157 patients)

	Assessments performed, n (%)	Foot problems identified from assessments performed, n (%)
Skin/nail	128 (81.5%)	126 (98.4%)
Vascular	121 (77.1%)	68 (56.2%)
Neurological	114 (72.6%)	66 (57.9%)
Structural	115 (73.2%)	106 (92.2%)
Biomechanical (static)	138 (87.9%)	131 (94.9%)
Biomechanical (dynamic)	105 (66.9%)	101 (96.2%)
Footwear/orthotic	144 (91.7%)	116 (80.6%)

### Treatments provided

Overall, the most common service provided to patients was foot-care education (*n* = 151, 96.2%), followed by general skin/nail care (*n* = 107, 68.2%) (Table 4). Over half of patients also received a footwear intervention during at least one appointment (*n* = 96, 61,1%), with almost two-thirds receiving orthoses (*n* = 64, 40.8%). Padding/offloading, wound care, and exercise prescription were less commonly provided (46.5, 20.4, and 15.3% of patients, respectively). Seventeen (10.8%) patients were also referred to other health practitioners, the most common being physiotherapists and general practitioners (5.1 and 3.8%, respectively).

**Table 4:** Treatments provided to patients who attended the Podiatric Rheumatology Clinic (*n* = 157 patients)

Education		151 (96.2%)
Skin/nail care		107 (68.2%)
Orthoses		64 (40.8%)
Footwear		96 (61.1%)
Wound care		32 (20.4%)
Padding/offloading		73 (46.5%)
Exercise prescription		24 (15.3%)
Referral^a^ (*n* = 17, 10.8%)	General practitioner	6 (3.8%)
	Rheumatologist	1 (0.7%)
	Physiotherapist	8 (5.1%)
	Occupational therapist	1 (0.7%)
	District health nurse	1 (0.7%)
	Imaging	1 (0.7%)

^a^1 patient was referred to two practitioners

Based on appointment-level data, foot-care education and skin/nail care were the most commonly provided interventions (85.0 and 84.5%, respectively) (Additional Table 2). Padding/offloading, footwear, and orthoses were provided during a similar proportion of appointments (15.0, 14.5, and 13.4%, respectively) Wound care and exercise prescription were the least commonly provided treatments (6.0 and 1.9% of appointments, respectively).

## Discussion

This is the first study focusing on podiatric service provision for people with inflammatory rheumatic diseases attending a specialist podiatric rheumatology clinic in Aotearoa New Zealand. The findings have shown that patients exhibit a wide range of foot problems, which often go beyond those they self-report. In addition, although education and general skin and/or nail care were the most common treatments provided to patients during regular follow-up appointments, most patients also received orthoses, footwear, padding and/or offloading at some point during their period of service provision.

Consistent with existing literature, this study has shown that people with inflammatory rheumatic diseases report a range of foot-related concerns, with foot pain and skin/nail problems among the most common, and neurovascular problems occurring less frequently [4, 7, 21–23]. However, the foot problems identified from podiatric assessments often went beyond the concerns reported by patients, with the majority of patients also exhibiting clinical evidence of biomechanical, structural, and footwear-related problems. These findings clearly highlight the importance of thorough podiatric clinical assessments to identify all factors contributing to the patients concerns, as well as patient education surrounding the impact of inflammatory rheumatic diseases on foot and lower limb pain and disability.

Results from this study have also demonstrated the range of foot-care interventions provided to people with inflammatory rheumatic diseases. Consistent with current guidelines and expert recommendations [14, 24–28], patients attending the AUT Podiatric Rheumatology Clinic received treatment of skin and nail problems, wound care, clinical padding, foot orthoses, and footwear prescription and advice. However, the most common intervention was foot-care education, provided during almost all appointments. Despite the absence of any formal published guidelines on education for rheumatology patients attending podiatry services [29, 30], foot-care education is recognised as a key intervention for people with inflammatory rheumatic diseases in improving patient knowledge and health outcomes [27, 31, 32]. In terms of overall service provision, the results from this study show promise in addressing the previously reported unmet need for provision of podiatric services to support people with inflammatory rheumatic diseases in Aotearoa New Zealand [18].

The current review also captured data relating to referrals, attendance and wait times. The majority of patients were referred from rheumatology services, with very few from other health providers, likely due to the paucity of specialist podiatric rheumatology clinics in Aotearoa New Zealand and the lack of awareness among potential referrers. In terms of attendance, a large proportion of patients referred to the AUT Podiatric Rheumatology Clinic did not attend an appointment. Existing research has highlighted a number of factors which may influence a patients decision to access foot-care, including a lack of patient awareness around the role of the podiatrist [33], perceived costs, as well as personal values and feeling that their feet are unimportant if they had been overlooked in clinical practice [7, 34, 35]. These barriers, which influence the patient’s decision to self-refer, suggest that patients should not be given the sole responsibility to seek podiatry services if they are to be delivered in an appropriate and timely manner [35]. Previous research has also highlighted that knowledge of the depth and value of podiatry practice by referring practitioners is lacking, and strategies that raise the profile of the profession, facilitate more streamlined pathways, and development of national clinical practice guidelines may improve uptake of podiatry services for patients with inflammatory rheumatic diseases [33, 36]. Finally, in reference to wait times, the relatively short wait times between referral and appointment observed in the current review are promising (median ~ 2 months). The majority of patients also attended regular four-monthly appointments which may support the importance of regular foot-care to prevent the development of new foot problems and address the changing concerns of the patient over time [14, 24–28].

Māori and Pacific peoples were under-represented among patients attending the AUT Podiatric Rheumatology Clinic relative to the proportion of these ethnic groups with rheumatic diseases in Aotearoa New Zealand. A 2018 report by Arthritis New Zealand (Kaiponapona Aotearoa) revealed the prevalence of rheumatic diseases was 1.24 times higher in Māori compared with non-Māori [19]. The low proportion of Māori patients attending podiatry services in the current study may be related to the location of the clinic in north Auckland, which has a relatively low Māori population compared to other regions in Aotearoa New Zealand [37]. These findings reflect the need for improved access to specialist podiatric rheumatology services to reduce inequities, which address referrer attitudes and behaviours, barriers related to attending clinics, and continued cultural safety training for students and practitioners [38–40]. Future work will evaluate how current service provision can be adapted to facilitate equitable access to podiatry services for patients with inflammatory rheumatic diseases across all ethnicity groups.

This study has some limitations. Firstly, although the student-led AUT Podiatric Rheumatology Clinic aims to offer a standard approach to service provision consistent with usual practice, appointment times are often slightly longer (~ 1 hour) which may allow for more in-depth assessments to be carried out compared to usual podiatric practice. Secondly, the retrospective nature of the current study meant data were extracted from clinical records that were not originally designed for research purposes and there is currently an absence of validated tools to categorise clinical record data based on service provision. However, the processes used to categorise this data have been explicitly outlined in the methods section to improve reproducibility. It should also be acknowledged that this review included data from 2020 through 2021 in which the Covid-19 pandemic and nationwide lockdowns would have influenced appointment numbers and time between appointments due to closure of the clinic. Finally, this review did not capture data related to the efficacy of podiatric services for people with inflammatory rheumatic diseases due to the absence of a standardised measure of treatment outcome used consistently across appointments. This will be the focus of future research.

## Conclusion

In conclusion, this study has shown that people with inflammatory rheumatic diseases present with a wide range of foot problems that go beyond general skin/nail care and education to include footwear, orthoses, other padding/offloading devices, wound care, exercise prescription and referrals. The results have also shown that a specialist podiatric rheumatology clinic dedicated to people with inflammatory rheumatic diseases can provide services targeted towards these foot problems.

## Supplementary Information

**Additional file 1: Table S1.** Baseline characteristics for referred patients who did not attend an appointment, in comparison with those who attended the Podiatric Rheumatology Clinic. **Table S2.** Service provision for patients who attended the Podiatric Rheumatology Clinic based on appointment level data^a^ (*n* = 1570 appointments).

## Data Availability

The datasets used and/or analysed during the current study are available from the corresponding author on reasonable request.

## Abbreviation

AUT: Auckland University of Technology

## References

[1] Walker-Bone K, Cooper C (2000). The spectrum of inflammatory rheumatic disorders. Best Pract Res Clin Rheumatol.

[2] Carter K, Lahiri M, Cheung PP, Santosa A, Rome K (2016). Footwear characteristics in people with inflammatory arthritis in Singapore. J Foot Ankle Res..

[3] Stewart S, Morpeth T, Dalbeth N, Vandal AC, Carroll M, Davidtz L, Mawston G, Otter S, Rome K (2016). Foot-related pain and disability and spatiotemporal parameters of gait during self-selected and fast walking speeds in people with gout: a two-arm cross sectional study. Gait Posture.

[4] Stewart S, Dalbeth N, Aiyer A, Rome K (2020). Objectively assessed foot and ankle characteristics in patients with systemic lupus erythematosus: a comparison with age- and sex-matched controls. Arthritis Care Res (Hoboken).

[5] Wilson O, Hewlett S, Woodburn J, Pollock J, Kirwan J (2017). Prevalence, impact and care of foot problems in people with rheumatoid arthritis: results from a United Kingdom based cross-sectional survey. J Foot Ankle Res..

[6] Patience A, Helliwell PS, Siddle HJ (2018). Focussing on the foot in psoriatic arthritis: pathology and management options. Expert Rev Clin Immunol.

[7] Wilson O, Kirwan J, Dures E, Quest E, Hewlett S (2017). The experience of foot problems and decisions to access foot care in patients with rheumatoid arthritis: a qualitative study. J Foot Ankle Res..

[8] Williams AE, Graham AS (2012). ‘My feet: visible, but ignored . . .’ A qualitative study of foot care for people with rheumatoid arthritis. Clin Rehabil.

[9] National Institute for Healh and Care Excellence. NICE guideline [NG100] Rheumatoid arthritis in adults: Management. 2020.

[10] Luqmani R, Hennell S, Estrach C, Basher D, Birrell F, Bosworth A, Burke F, Callaghan C, Candal-Couto J, Fokke C, Goodson N, Homer D, Jackman J, Jeffreson P, Oliver S, Reed M, Sanz L, Stableford Z, Taylor P, Todd N, Warburton L, Washbrook C, Wilkinson M, British Society for Rheumatology, British Health Professionals in Rheumatology Standards, Guidelines and Audit Working Group (2009). British Society for Rheumatology and British health professionals in rheumatology guideline for the management of rheumatoid arthritis (after the first 2 years). Rheumatology (Oxford).

[11] SIGN. Management of early rheumatoid arthritis: a national clinical guideline. Edinburgh; 2000.

[12] National Institute for Health and Care Excellence. NICE guideline [NG100]: Rheumatoid arthritis in adults: management. 2020.

[13] Luqmani R, Hennell S, Estrach C, Birrell F, Bosworth A, Davenport G, Fokke C, Goodson N, Jeffreson P, Lamb E, Mohammed R, Oliver S, Stableford Z, Walsh D, Washbrook C, Webb F, British Society for Rheumatology, British Health Professionals in Rheumatology Standards, Guidelines and Audit Working Group (2006). British Society for Rheumatology and British health professionals in rheumatology guideline for the Management of Rheumatoid Arthritis (the first two years). Rheumatology..

[14] Network SIG. Management of early rheumatoid arthritis. A National Clinical Guideline. Edinburgh: Royal College of Physicians of Edinburgh; 2000.

[15] Williams AE, Bowden AP (2004). Meeting the challenge for foot health in rheumatic diseases. Foot.

[16] Hendry GJ, Gibson KA, Pile K, Taylor L, Du Toit V, Burns J (2013). "They just scraped off the calluses": a mixed methods exploration of foot care access and provision for people with rheumatoid arthritis in South-Western Sydney. Australia J Foot Ankle Res.

[17] Hendry GJ, Gibson KA, Pile K, Taylor L, du Toit V, Burns J, Rome K (2013). Provision of foot health services for people with rheumatoid arthritis in New South Wales: a web-based survey of local podiatrists. J Foot Ankle Res..

[18] Rome K, Gow PJ, Dalbeth N, Chapman JM (2009). Clinical audit of foot problems in patients with rheumatoid arthritis treated at counties Manukau District health board, Auckland. New Zealand J Foot Ankle Res.

[19] Arthritis New Zealand. The economic cost of arthritis in 2018. 2018.

[20] Chapman LS, Redmon AC, Landorf KB, Rome K, Keenan A, Waxman R, et al. A survey of foot orthoses prescription habits amongst podiatrists in the UK, Australia and New Zealand. J Foot Ankle Res. 2018;11(1):64. 10.1186/s13047-018-0304-z. 10.1186/s13047-018-0304-z PMC6258496 30505351

[21] Hyslop E, McInnes IB, Woodburn J, Turner DE (2010). Foot problems in psoriatic arthritis: high burden and low care provision. Ann Rheum Dis.

[22] Sari-Kouzel H, Hutchinson CE, Middleton A, Webb F, Moore T, Griffin K, Herrick AL (2001). Foot problems in patients with systemic sclerosis. Rheumatology (Oxford).

[23] Stewart S, Dalbeth N, Vandal AC, Rome K (2015). Characteristics of the first metatarsophalangeal joint in gout and asymptomatic hyperuricaemia: a cross-sectional observational study. J Foot Ankle Res..

[24] Conditions NCCfC. Rheumatoid arthritis: national clinical guideline for management and treatment in adults. 2009. 21413195

[25] Podiatry Rheumatic Care Association (2008). Standards of care for people with musculoskeletal foot health problems.

[26] Arthritis and Musculoskeletal Aliance. Standard of care for people with inflammatory arthritis. 2004.

[27] Williams AE, Davies S, Graham A, Dagg A, Longrigg K, Lyons C, Bowen C, North West Clinical Effectiveness Group for the Foot in Rheumatic Diseases (NWCEG) (2011). Guidelines for the management of the foot health problems associated with rheumatoid arthritis. Musculoskeletal Care.

[28] Huijbrechts EJ, Dekker J, Tenten-Diepenmaat M, Gerritsen M, van der Leeden M (2021). Clinical guidance for podiatrists in the management of foot problems in rheumatic disorders: evaluation of an educational programme for podiatrists using a mixed methods design. J Foot Ankle Res.

[29] Graham AS, Hammond A, Williams AE (2012). Foot health education for people with rheumatoid arthritis: the practitioner's perspective. J Foot Ankle Res..

[30] Graham AS, Williams AE (2016). Foot health education provision for people with rheumatoid arthritis-an online survey of UK podiatrists' perceptions. J Foot Ankle Res..

[31] Abourazzak F, El Mansouri L, Huchet D, Lozac'hmeur R, Hajjaj-Hassouni N, Ingels A (2009). Long-term effects of therapeutic education for patients with rheumatoid arthritis. Joint Bone Spine.

[32] Masiero S, Boniolo A, Wassermann L, Machiedo H, Volante D, Punzi L (2007). Effects of an educational-behavioral joint protection program on people with moderate to severe rheumatoid arthritis: a randomized controlled trial. Clin Rheumatol.

[33] McCulloch L, Borthwick A, Redmond A, Edwards K, Pinedo-Villanueva R, Prieto-Alhambra D, Judge A, Arden NK, Bowen CJ (2018). UK podiatrists’ experiences of podiatry services for people living with arthritis: a qualitative investigation. J Foot Ankle Res..

[34] de Souza S, Williams R, Lempp H (2016). Patient and clinician views on the quality of foot health care for rheumatoid arthritis outpatients: a mixed methods service evaluation. J Foot Ankle Res..

[35] Blake A, Mandy PJ, Stew G (2013). Factors influencing the patient with rheumatoid arthritis in their decision to seek podiatry. Musculoskeletal Care..

[36] Carter K, Cheung PP, Rome K, Santosa A, Lahiri M (2017). Increasing podiatry referrals for patients with inflammatory arthritis at a tertiary hospital in Singapore: a quality improvement project. Foot (Edinb).

[37] New Zealand Statistics Tatauranga Aotearoa. Ethnic group (detailed total response - level 3) by age and sex, for the census usually resident population count, 2006, 2013, and 2018 Censuses 2022.

[38] Palmer SC, Gray H, Huria T, Lacey C, Beckert L, Pitama SG (2019). Reported Māori consumer experiences of health systems and programs in qualitative research: a systematic review with meta-synthesis. Int J Equity Health.

[39] Sheridan NF, Kenealy TW, Connolly MJ, Mahony F, Barber PA, Boyd MA, Carswell P, Clinton J, Devlin G, Doughty R, Dyall L, Kerse N, Kolbe J, Lawrenson R, Moffitt A (2011). Health equity in the New Zealand health care system: a national survey. Int J Equity Health.

[40] Ryan D, Grey C. Tofa Saili: a review of evidence about health equity for Pacific peoples in New Zealand. Wellington: Pacific Perspectives Ltd; 2019.

