# Supplementary material for: An evaluation of podiatry service use for people with inflammatory rheumatic diseases: a review of a rheumatology podiatry clinic in Aotearoa New Zealand
Source: J Foot Ankle Res. 2022 May 16;15:36. doi: 10.1186/s13047-022-00542-7 (PMC9108704; doi:10.1186/s13047-022-00542-7)
Supplement: Supplementary file 1 — Additional file 1: Table S1. Baseline characteristics for referred patients who did not attend an appointment, in comparison with those who attended the Podiatric Rheumatology Clinic. Table S2. Service provision for patients who attended the Podiatric Rheumatology Clinic based on appointment level dataa (n = 1570 appointments). [file 13047_2022_542_MOESM1_ESM.docx]

| **Additional Table S1.** Baseline characteristics for referred patients who did not attend an appointment, in comparison with those who attended the Podiatric Rheumatology Clinic | | | | |
| --- | --- | --- | --- | --- |
|  | | **Patients who attended an appointment (n = 157)** | **Referred patients who did not attend an appointment (n = 52)** | ***P*** |
| Age group, years, n (%) | <10 | 0 (0.0%) | 0 (0.0%) | 0.08 |
|  | 11-20 | 1 (0.6%) | 0 (0.0%) |  |
|  | 21-30 | 5 (3.2%) | 4 (7.7%) |  |
|  | 31-40 | 12 (7.6%) | 4 (7.7%) |  |
|  | 41-50 | 17 (10.8%) | 5 (9.6%) |  |
|  | 51-60 | 29 (18.5%) | 20 (38.5%) |  |
|  | 61-70 | 40 (25.5%) | 9 (17.3%) |  |
|  | 71-80 | 42 (26.8%) | 8 (15.4%) |  |
|  | 81-90 | 11 (7.0%) | 2 (3.8%) |  |
|  | 90+ | 0 (0.0%) | 0 (0.0%) |  |
| Gender, n (%) | Male | 36 (22.9%) | 14 (26.9%) | 0.56 |
|  | Female | 121 (77.1%) | 38 (73.1%) |  |
| Ethnicity, n (%) | NZ Māori | 5 (3.2%) | 2 (3.8%) | 0.92 |
|  | Pacific peoples | 4 (2.5%) | 1 (1.9%) |  |
|  | European | 116 (73.9%) | 32 (61.5%) |  |
|  | Asian | 22 (14.0%) | 5 (9.6%) |  |
|  | Middle Eastern/Latin American/African | 2 (1.3%) | 0 (0.0%) |  |
|  | Not reported | 8 (5.1%) | 12 (23.1%) |  |
| Rheumatic disease^a^, n (%) | Gout | 8 (5.1%) | 2 (3.8%) | 0.031 |
|  | Juvenile idiopathic arthritis | 4 (2.6%) | 2 (3.8%) |  |
|  | Other spondylarthritis | 8 (5.1%) | 6 (11.5%) |  |
|  | Psoriatic arthritis | 14 (8.9%) | 4 (7.7%) |  |
|  | Rheumatoid arthritis | 123 (78.3%) | 34 (65.4%) |  |
|  | Scleroderma | 2 (1.3%) | 3 (5.8%) |  |
|  | Systemic lupus erythematosus | 7 (4.5%) | 0 (0.0%) |  |
|  | Other | 6 (3.8%) | 2 (3.9%) |  |
| Disease duration, years, mean (SD), range | | 19.6 (14.6), 0.5 – 79.0 | 19.9 (15.7), 1 – 44 | 0.23 |
| Referrer, n (%) | Rheumatologist | 89 (56.7%) | 52 (100.0%) | - |
|  | Self-referred | 30 (19.1%) | 0 (0.0%) | - |
|  | General practitioner | 9 (5.7%) | 0 (0.0%) | - |
|  | Other | 9 (5.7%) | 0 (0.0%) | - |
|  | Unknown | 20 (12.7%) | 0 (0.0%) | - |
| Reasons for referral^b^, n (%) | General skin/ nail care | - | 16 (30.8%) | - |
|  | Foot pain | - | 23 (44.2%) | - |
|  | Footwear/ Orthotics | - | 22 (42.3%) | - |
|  | Foot deformity | - | 21 (40.4%) | - |
|  | Other | - | 8 (15.4%) | - |
| ^a^3 patients had 2 rheumatic diseases; ^b^23 patients had 1 reason for referral, 22 patients had 2 reasons for referral, 5 patients had 3 reasons for referral, 2 patients had 4 reasons for referral | | | | |

| **Additional Table S2.** Service provision for patients who attended the Podiatric Rheumatology Clinic based on appointment level data^a^ (n = 1570 appointments) | | | |
| --- | --- | --- | --- |
| Presenting complaint^b^, n (%) | General skin/nail care | | 1273 (81.1%) |
|  | Foot pain | | 587 (37.4%) |
|  | Footwear/orthotics | | 273 (17.4%) |
|  | Back and/or lower limb pain | | 36 (2.3%) |
|  | Foot deformity | | 16 (1.0%) |
|  | Neurological symptoms | | 17 (1.1%) |
|  | Wounds/ulcers | | 12 (0.8%) |
|  | Recent falls/balance issue | | 41 (2.6%) |
|  | Other | | 44 (2.8%) |
| Assessments performed, n (%) | Skin/nail | | 1403 (89.4%) |
|  | Vascular | | 1023 (65.2%) |
|  | Neurological | | 518 (33.0%) |
|  | Structural | | 929 (59.2%) |
|  | Biomechanical (static) | | 656 (41.8%) |
|  | Biomechanical (dynamic) | | 362 (23.1%) |
|  | Footwear/orthotic | | 934 (59.5%) |
|  | Other | | 1 (0.1%) |
| Foot problems identified^c^, n (%) | Skin/nail | | 1395 (88.9%) |
|  | Vascular | | 594 (37.8%) |
|  | Neurological | | 252 (16.1%) |
|  | Structural | | 923 (58.8%) |
|  | Biomechanical (static) | | 619 (39.4%) |
|  | Biomechanical (dynamic) | | 328 (20.9%) |
|  | Footwear/orthotic | | 480 (30.6%) |
|  | Falls risk | | 8 (0.5%) |
|  | Other | | 2 (0.1%) |
| Treatments provided, n (%) | Education | | 1334 (85.0%) |
|  | Skin/nail care | | 1327 (84.5%) |
|  | Orthoses | | 211 (13.4%) |
|  | Footwear | | 277 (14.5%) |
|  | Wound care | | 94 (6.0%) |
|  | Padding/offloading | | 235 (15.0%) |
|  | Exercise prescription | | 30 (1.9%) |
|  | Referral  (n = 20, 1.3%) | General practitioner | 8 (0.5%) |
|  |  | Rheumatologist | 1 (0.1%) |
|  |  | Physiotherapist | 8 (0.5%) |
|  |  | Occupational therapist | 1 (0.1%) |
|  |  | District health nurse | 1 (0.1%) |
|  |  | Imaging | 1 (0.1%) |
| ^a^Appointment-level data refers to the number of appointments in which the complaint, assessments, foot problems, and treatments were recorded; ^b^Reported by the patient during the appointment; ^c^Identified from assessments undertaken during the appointment. | | | |
